# Interspecific Hybridization Barrier Between *Paeonia ostii* and *P. ludlowii*

**DOI:** 10.3390/plants14071120

**Published:** 2025-04-03

**Authors:** Yingzi Guo, Yan Zhang, Yanli Wang, Guodong Zhao, Wenqing Jia, Songlin He

**Affiliations:** 1 College of Landscape Architecture and Art, Henan Agricultural University, Zhengzhou 450002, China; guoyz2017@126.com; 2 College of Horticulture and Landscape Architecture, Henan Institute of Science and Technology, Xinxiang 453003, China; mint_19@163.com (Y.Z.); yanliwang0218@yeah.net (Y.W.); 3 Luoyang National Peony Gene Bank, Luoyang 471099, China; zhaoguodong2025@163.com

**Keywords:** interspecific hybridization, tree peony, distant hybridization, pollen–pistil incompatibility, KCl solution treatment

## Abstract

*Paeonia ludlowii* is a threatened and valuable germplasm in the cultivated tree peony gene pool, with distinctive traits such as tall stature, pure yellow flowers, and scarlet foliage in autumn. However, the crossability barrier limits gene transfer from *P. ludlowii* to cultivated tree peony. Therefore, our study investigated the reasons for the lack of crossability between *P. ludlowii* and *Paeonia ostii* ‘Fengdan’. Distant cross pollination (DH) resulted in the formation of many calloses at the ends of the pollen tubes, which grew non-polar, twisted, entangled, and often stopped in the style. Pollen tubes elongated the fastest in self-pollination (CK), and pollen tubes elongated faster and fewer pollen tube abnormalities were observed in stigmas treated with KCl solution before pollination (KH) than in DH. During pollen–pistil interactions, the absence of stigma exudates, high levels of H_2_O_2_, O_2_^−^, MDA, ^•^OH, ABA, and MeJA, and lower levels of BR and GA_3_ may negatively affect pollen germination and pollen tube elongation in the pistil of *P. ostii* ‘Fengdan’. Pollen tubes in CK and KH penetrated the ovule into the embryo sac at 24 h after pollination, whereas only a few pollen tubes in DH penetrated the ovule at 36 h after pollination. Pre-embryo abnormalities and the inhibition of free nuclear endosperm division resulted in embryo abortion in most of the fruits of DH and many fruits of KH, which occurred between 10 and 20 days after pollination, whereas embryos in CK developed well. Early embryo abortion and endosperm abortion in most of the fruits of DH and KH led to seed abortion. Seed abortion in KH and DH was mainly due to an insufficient supply of auxins and gibberellins and lower content of soluble protein and soluble sugars. The cross failure between *P. ludlowii* and *P. ostii* ‘Fengdan’ is mostly caused by a pre-fertilization barrier. KH treatment can effectively promote pollen tube growth and facilitate normal development of hybrid embryos. These findings provide new insights into overcoming the interspecific hybridization barrier between cultivated tree peony varieties and wild species.

## 1. Introduction

The presence of a narrow genetic base in cultivated species is one of the major barriers for breeders to develop varieties for high stress resistance and high quality [1]. Distant crosses are essential in crop development because they can be used to incorporate genes from wild relatives into cultivated crops for high resistance and yield improvement in cultivated plant species [2]. The exploitation of untapped genes from wild genetic resources is the best hope for future agricultural improvement [3]. Distant hybridization is a widely used chromosome manipulation method for crop improvement [4]. ‘Xiaoyan 6’ is an exceptional type of wheat with high resistance to stripe rust. It was created by distant hybridization with exogenous genes from *Elytrigia elongata* in the 1970s. It has been widely farmed in China for the past three decades [5]. ‘Xiaohuyang-1’ is an outstanding cultivar created by distant hybridization of *Populus* sections Tacamahaca and Turanga [5]. Interspecific crosses between *Tulipa gesneriana* genotypes and other Tulipa species have been widely used in Tulipa to expand the commercial range with desired traits [6].

Distant crosses have often failed for a variety of reasons such as barriers before and after fertilization, which ultimately prevent the production of hybrid zygotes [7,8]. Understanding the mechanisms responsible for the lack of affinity in long-distance hybridization is important for successfully obtaining hybrid zygotes/hybrid embryos by overcoming these barriers [9]. Distant hybridization pollination often fails because of obstacles before fertilization, such as poor pollen germination, sluggish pollen tube growth and inability to reach the embryo sac [7,10,11]. The excessive accumulation of callose leads to incompatible hybridization reactions in rice [12].

O_2_^−^, H_2_O_2_ and ^•^OH in pistil cells affect pollen germination, polar growth of the pollen tube and fertilization [13,14,15,16,17]. Higher levels of H_2_O_2_ would damage cell structures and reproductive activities [18]. Overproduction of ROS in the pistil was a major cause of hybridization failure between *B. napus* and *B. oleracea* [19]. The germination of peony pollen is positively related to superoxide dismutase (SOD) levels and negatively related to malondialdehyde (MDA) levels [20]. Excessive ROS in stigma cells led to self-incompatibility in Brassicaceae species [21]. High ROS levels inhibited pollen germination [22]. Gibberellic acid (GA_3_) promoted pollen germination and growth in the post-pollination process of orchids [23]. High levels of trans-zeatin-riboside (ZR) and 3-indoleacetic acid (IAA) are beneficial for early and mid-stage zygotic development [24]. High levels of abscisic acid (ABA) were the main cause of self-incompatibility in the study of tea plants [25]. Lower levels of ABA and brassinolide (BR) are major contributors to the establishment of intersubgeneric hybridization obstacles in water lilies [21]. Methyl jasmonate (MeJA) may be one of the main factors affecting self-incompatibility in pear [26]. Zeatin riboside (ZR) and ABA are the main endogenous hormones involved in plant fertilization [25,27,28].

Seed abortion after fertilization can also have serious implications for the use of good germplasm resources in ornamental plants. Embryo abortion may be attributed to endosperm degeneration or partial sterility in hybrids [29,30,31]. Fertilization can still occur in interspecific crosses, but subsequent embryo abortion can be attributed to abnormalities in endosperm development [32,33]. The degradation or death of zygotes is one of the main causes of post-fertilization barriers [8]. Abnormal IAA levels can disrupt the hormonal balance of the ovule and cause embryo abortion [25,34]. Ethylene has been proposed as a potential factor in the induction of ovule abortion in maize and wheat [35]. In banana, although ovule fertilization occurs, the majority experience abortion, particularly at the proximal end of the ovary [36].

**Figure 1:**
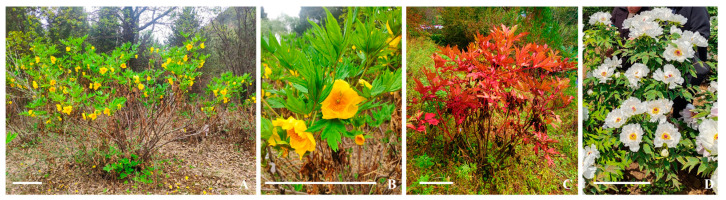
Paeonia ludlowii and *P. ostii* ‘Fengdan’. (**A**–**C**) *P. ludlowii*; (**D**) *P. ostii* ‘Fengdan’; bar = 50 cm.

*Paeonia ostii* ‘Fengdan’ is widely cultivated in East Asia for its vital medicinal values, excellent ornamental quality, and many superior agronomic traits such as tall stature, moisture and heat resistance, and good seed set. It is one of the most important founding parents in tree peony and has played a crucial role in Chinese tree peony breeding programs [37]. *Paeonia ludlowii* is an endangered species found only in Xizang Province, China. It is an important tree peony genetic resource for its tall stature, yellow flowers and scarlet leaves [38] in autumn (Figure 1A–C), but its tolerance to high temperatures is low, whereas *P. ostii* ‘Fengdan’ has exceptional tolerance to high temperatures (Figure 1D).

In contrast to *P. delavayi*, the use of the excellent ornamental genes of *P. ludlowii* in tree peony improvement has not been well documented on a global scale, and the excellent ornamental genes of *P. ludlowii* have not been transferred to the central plain tree peony cultivar [39] due to reasons such as lack of affinity in crossbreeding. Interspecific wide hybridization has been the main means of transferring desirable traits from *P. ludlowii* to tree peony cultivars. Little is known about the physiological and molecular mechanisms regulating the pollen–pistil interactions in distant hybridization between *P. ostii* ‘Fengdan’ and *P. ludlowii*. In our previous study, we found apparent pollen–pistil incompatibility in the interspecific hybrid between *P. ostii* ‘Fengdan’ and *P. ludlowii*, and the incompatibility phenomena greatly hindered the use of excellent *P. ludlowii* genes. Meanwhile, we observed that treatment with KCl solution was effective in increasing the fruiting rate of interspecific hybrids. In this study, interspecific hybridization was carried out between *P. ostii* ‘Fengdan’ and *P. ludlowii*. Therefore, the objectives of this study were to (1) analyze the cytological causes of poor affinity in interspecific crosses, (2) identify the types of interspecific crosses that are not affinitive, and (3) investigate the reasons why KCl solution improves the fertility of interspecific crosses. Our findings provide technical support for artificial pollination breeding of tree peonies.

## 2. Results

### 2.1. Morphology of Papilla Cell in Stigma and Pollen Germination

**Figure 2:**
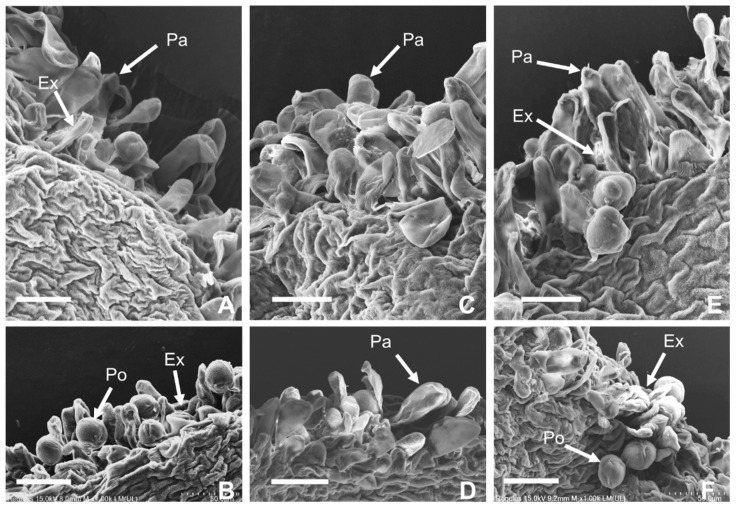
Effect of different pollination methods on stigma papilla cell morphology and pollen germination. (**A**) Stigmas at 2 h after pollination in control (CK); (**B**) stigmas at 12 h after pollination in CK; (**C**) stigmas at 2 h after pollination in distant hybrid pollination (DH); (**D**) stigmas at 12 h after pollination in DH; (**E**) stigmas at 2 h after pollination in stigmas treated with KCl solution before pollination (KH); (**F**) stigmas at 2 h after pollination in KH; Pa: papilla; Ex: exudate; Po: pollen; bar = 50 μm.

At 2 h after pollination, the stigma papilla cells appeared irregularly shrunken and a certain amount of exudate was observed in CK; in DH, the papilla cells became slightly more swollen and no exudate was observed (Figure 2), and in KH, the papilla cells were fuller and more laminar exudate on the stigma was observed. At 12 h after pollination, a large amount of germinated pollen was accompanied by a large amount of exudate on CK and KH stigmas, while only a small amount of germinated pollen was observed in DH, and the majority of stigma papilla cells were in a filling state.

### 2.2. Pollen Germination and Growth in Pistil

**Figure 3:**
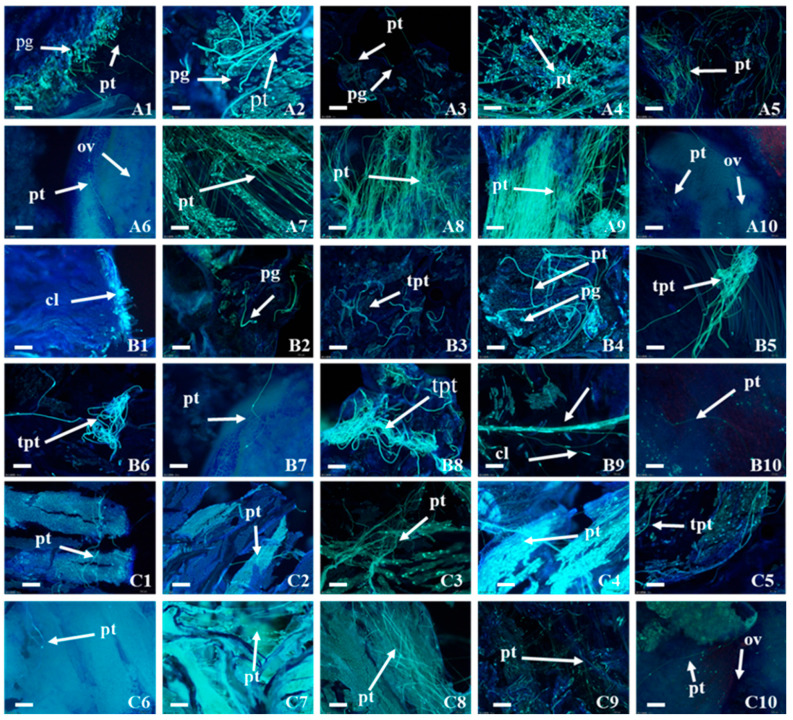
Pollen tube growth in control (CK), stigmas treated with KCl solution before pollination (KH) and distant hybrid pollination (DH) treatments. (**A1**–**A10**) Pollen tube growth at 2, 4, 6, 8, 12, 24, 36, 48, 72 and 96 h after pollination in CK; (**B1**–**B10**) pollen tube growth at 2, 4, 6, 8, 12, 24, 36, 48, 72 and 96 h after pollination in DH treatment; (**C1**–**C10**) pollen tube growth at 2, 4, 6, 8, 12, 24, 36, 48, 72 and 96 h after pollination in KH treatment; pg: pollen grain; pt: pollen tube; cl: callose; tpt: twisted pollen tube; bar = 100 μm.

Significant differences were observed in the pollen germination in the CK, DH and KH treatments. At 2 h post-pollination, a significant amount of pollen had already germinated in CK and KH, respectively, but very little pollen germination was observed in DH at 4 h post-pollination. Large pollen tubes grew and penetrated the embryo sac into the ovules at 24 h in CK. At 6 h post-pollination, large amounts of callose, which could prevent pollen tubes from penetrating the stigma surface, were observed on the stigmas in DH, pollen tubes were distorted and tended to twist (Figure 3B4–B8); at 12–24 h post-pollination, majority of pollen tubes changed from curved growth to tangled growth, growing disorderly and directionless in DH. At 36 h post-pollination, only a few pollen tubes penetrated the stylar transmitting tissue into the embryo sac; at 72 h post-pollination, swelling occurred at the ends of the pollen tubes, causing most of pollen tubes to stop growing in the style; at 96 h post-pollination, fewer pollen tubes still grew continuously, but remained on the surface of the ovary (Figure 3B8–B10). Compared with DH, the pollen germination rate and the number of pollen tubes were significantly increased in KH, but significantly lower than the control. Most of the pollen tubes had normal morphology when growing through the stylar transmitting tissue and tangling or swelling at the ends of the pollen tubes were not found during the growth process (Figure 3C1–C10). The time for the pollen tube to reach the ovule was also delayed by about 4 h compared with CK. Overall, the number of pollen tubes entering into the ovules was in the following order: CK > KH > DH.

### 2.3. Seed Setting Rate

**Figure 4:**
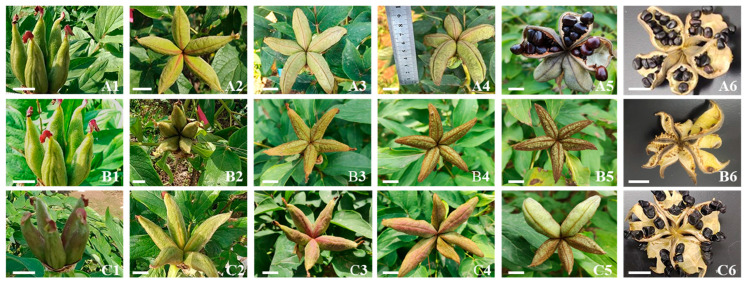
Fruit development in control (CK), stigmas treated with KCl solution before pollination (KH) and distant hybrid pollination (DH) treatments. (**A1**–**A6**) Fruits at 10, 30, 45, 60, 90 and 120 days after pollination in CK treatment; (**B1**–**B6**) fruits at 10, 30, 45, 60, 90 and 120 days after pollination in DH treatment; (**C1**–**C6**) fruits at 10, 30, 45, 60, 90 and 120 days after pollination in KH treatment; bar = 2 cm.

There were significant differences in the development of polymerized follicle ovaries in the CK, KH and DH treatments. At 10 days after pollination, there was no significant change in the development of polymerized follicle ovaries in the CK, KH and DH treatments. At 30 days after pollination, except for the DH treatment, the polymerized follicles of CK and KH began to open (Figure 4(A2,B2,C2)), and at 45–90 days after pollination, most of the follicle ovaries in the DH treatment stopped developing and finally formed the smallest fruits (Figure 4(B3–B5)). In the KH treatment, nearly two-thirds of the ovaries stopped developing, while the other half continued to develop and swell (Figure 4(C2–C4)), gradually developing into small- to medium-sized fruits at near maturity (Figure 4C5), and in the case of the CK treatment, the follicle ovaries developed normally, swelled (Figure 4(A2–A5)), and continued to develop into large, ripe fruits by 90 to 110 days after pollination (Figure 4(A5,A6)). The rate of fruit set in the KH treatments was about 1.5 times higher than in the DH treatments (Table 1). More fruits were produced in KH than in DH. The number of plump seeds in the KH treatments was about 15–20 times higher than in the DH treatments. Although the average number of seeds per fruit in KH was only 2.28 less than that in DH treatment, the number of plump seeds was only 12.04% of that in CK. The signal follicle weight of KH fruits increased compared to DH fruits. The average weight of fruits in CK was the heaviest, about 5.38 times that in KH. The average signal follicle weight of the KH pollinated fruits was found to be 2.02 times that of DH pollinated fruits (Table 1). This indicates that there is significant embryo abortion in crosses between *P. ostii* ‘Fengdan’ and *P. ludlowii*.

### 2.4. Hormone Contents

**Figure 5:**
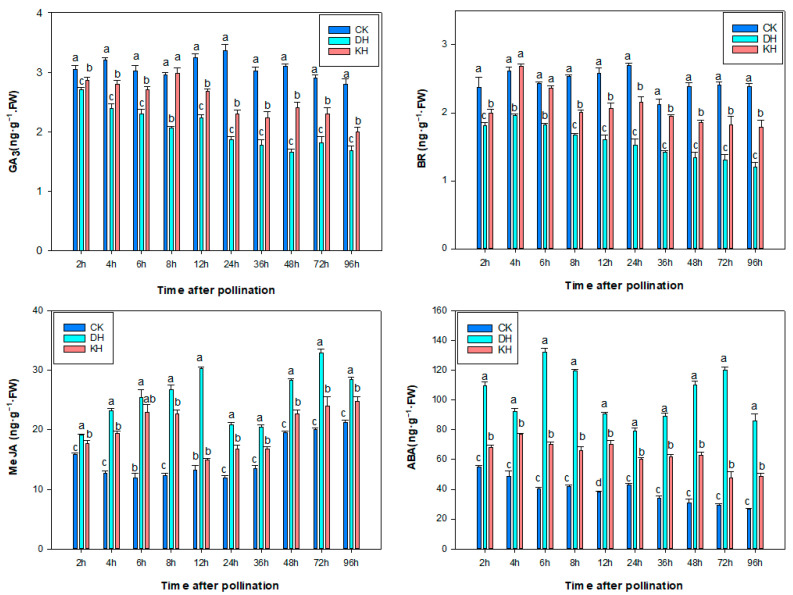
Effects of control (CK), stigmas treated with KCl solution before pollination (KH) and distant hybrid pollination (DH) treatments on endogenous hormone levels. Error bars represent mean ± standard deviation (SD); different lowercase letters indicate significant differences at *p* < 0.05.

To understand whether phytohormone levels could regulate the pollination or result in pre-fertilization barrier in tree peony, styles were collected in CK, KH and DH at 0, 2, 4, 6, 8, 12, 24, 36, 48, 72, 96 h after pollination, and the concentrations of ABA, GA_3_, BR and MeJA were measured. From 2 to 4 h after pollination, MeJA and ABA levels were significantly higher in DH than those in CK and KH. Pollen grain germination in CK and KH was observed at 2 h after pollination, whereas pollen grain germination in DH occurred at about 4–6 h after pollination (Figure 5), indicating that MeJA and ABA levels were related to pollen germination in DH. The germination of pollen grains in DH was slightly delayed compared to the CK and KH, possibly due to the higher levels of MeJA and ABA (Figure 5). The levels of ABA were significantly higher in DH than in CK and KH at 6–12 h after pollination, but these levels rapidly decreased in DH at 24 h after pollination. At 6–12 h, the pollen tubes in DH treatment showed obvious distortion and tangling in their growth in DH, while the pollen tube grew towards the embryo sac polarity, and no twisting or tangling of the pollen tube was found in CK and KH. This indicated that ABA was correlated with the polar growth of the pollen tube in the styles of DH (Figure 5). From 6 to 96 h after pollination, the levels of GA_3_, BR were significantly higher in CK and KH than those in DH, while the levels of MeJA and ABA were significantly lower in CK and KH than those in DH. During this period, more rapid elongation of pollen tubes in CK was observed compared to that in DH and KH, and the growth rate of pollen tubes in KH treatment was significantly higher than that in DH treatment. This indicated that higher levels of GA_3_ and BR contributed to pollen tube growth.

### 2.5. SOD Activity, ROS and MDA Content

**Figure 6:**
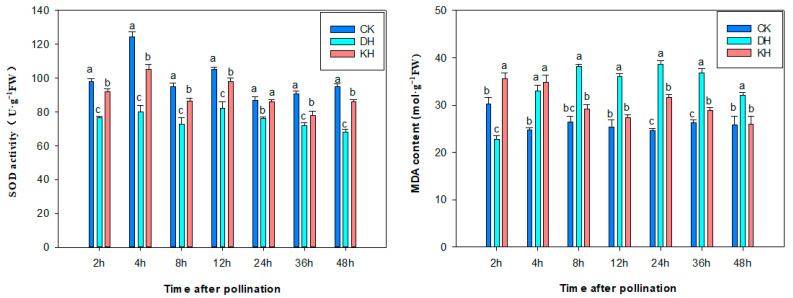
Changes in SOD activity and MDA levels in control (CK), stigmas treated with KCl solution before pollination (KH) and distant hybrid pollination (DH) treatments after pollination. Error bars represent mean ± standard deviation (SD); different lowercase letters indicate significant differences at *p* < 0.05.

SOD activities in CK, DH and KH treatments were significantly different at different times after pollination. From 2 h to 4 h post-pollination, pollen germinated and grew rapidly in CK and KH treatments, whereas pollen germination was not observed in DH treatment. SOD activity increased significantly in CK and KH treatments and SOD activities reached 124.3 and 105.2 U·g^−1^ at 4 h, respectively, and remained at a relatively high level thereafter. The SOD activity of DH pistils also increased from 2 to 4 h, reaching a peak of 80.1 U·g^−1^ at 4 h, when some pollen germination was observed on the stigma of DH treatment, and the SOD activity gradually decreased following this (Figure 6). This suggests that high SOD activity contributes to the affinity between pollen and stigma.

The MDA content of pistils in the CK, DH and KH treatments was significantly different at different times after pollination: in the CK treatment, the MDA content of pistils decreased significantly from 2 to 4 h after pollination (*p* < 0.05), reaching a minimum of 24.76 μmol·g^−1^ at 4 h. At this time, the pollen rapidly germinated and grew, and then the MDA content stayed a stable level, at which time the pollen tube reached the ovule and fertilization occurred (Figure 6). The MDA content in the pistils of the KH treatment decreased significantly (*p* < 0.05) from 2 to 12 h after pollination, reaching a minimum of 27.38 μmol·g^−1^ at 12 h while the pollen tube continued to grow, and then the MDA content increased and decreased. The MDA content in the pistils of the DH treatment increased from 2 to 4 h post-pollination. During the interval, the pollen did not germinate, and then the MDA content began to increase significantly (*p* < 0.05) after 4 h of pollination. During this period, some of the pollen could be seen to germinate, and then MDA remained at a high level from 8 to 36 h. From 2 to 4 h after pollination, the pollen in DH treatment did not germinate and the MDA content of pistils increased. At 4 h after pollination, some pollen grains were seen to germinate, while the MDA content started to increase significantly (*p* < 0.05), and it remained high for 8 to 36 h. During this interval, the twisted pollen tubes appeared in the stigmas and styles, accompanied by callus deposition, and pollen tube growth was blocked.

**Figure 7:**
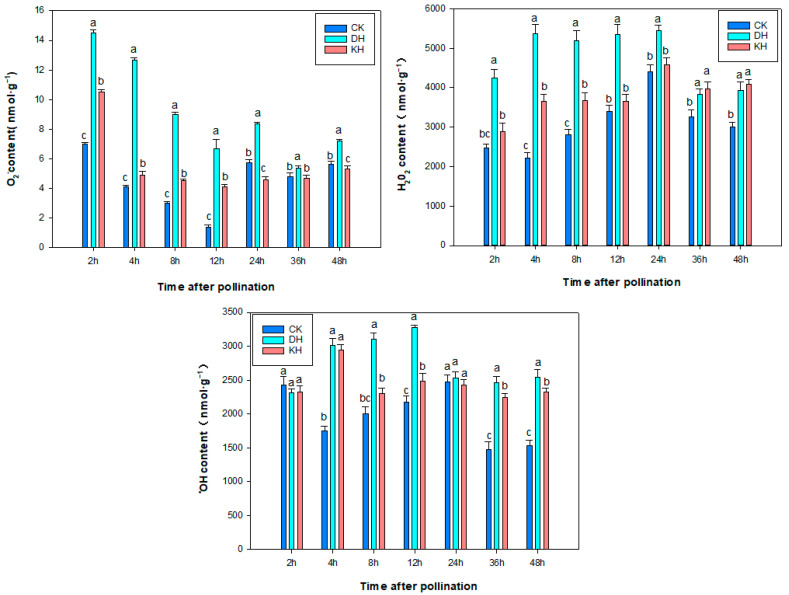
Changes in stigmatic ROS content in control (CK), stigmas treated with KCl solution before pollination (KH) and distant hybrid pollination (DH) treatments after pollination. Error bars represent mean ± standard deviation (SD); different lowercase letters indicate significant differences at *p* < 0.05.

To determine whether ROS (O_2_^−^, H_2_O_2_ and ^•^OH) are involved in pre-fertilization barrier response between *P. ludlowii* and *P. ostii* ‘Fengdan’, we measured the contents of O_2_^−^, H_2_O_2_ and ^•^OH of stigmas in CK, DH and KH at 2 h, 4 h, 8 h, 12 h, 24 h, 36 h and 48 h post-pollination. The O_2_^−^ and H_2_O_2_ activities of stigmas in DH were 14.2 nmol·g^−1^ and 4200 nmol·g^−1^ in CK at 2–4 h after pollination, about 2-fold that of stigmas in CK, which were significantly higher than those of stigmas in KH (Figure 7). This was in contrast to the responses of pollen germination in CK, DH and KH stigmas. The O_2_^−^ and H_2_O_2_ activities of stigmas in DH were higher than those in CK and KH 4–24 h after pollination, and at this time, twisted and entangled pollen tubes were observed in DH. This suggests that elevated O_2_^−^ and H_2_O_2_ were detrimental to pollen tube growth. No significant difference in stigmatic ^•^OH content was found between CK, DH and KH at 2 h after pollination. At 4–36 h after pollination, the ^•^OH content in CK was lower than that in KH and DH at all-time points except 24 h. This suggests that the high level of stigmatic O_2_^−^ and H_2_O_2_ may lead to the response of pre-fertilization barriers in DH.

### 2.6. Embryo Development Observation of Anatomy During Embryo Development

**Figure 8:**
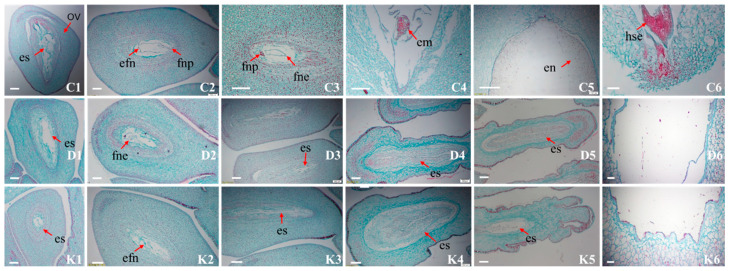
Morphological observation of ovule development in control (CK), stigmas treated with KCl solution before pollination (KH) and distant hybrid pollination (DH) treatments. (**C1**–**C6**) Ovules in CK at 5, 10, 20, 30, 40 and 50 days after pollination, respectively; (**D1**–**D6**) ovules in DH treatments at 5, 10, 20, 30, 40 and 50 days after pollination, respectively; (**K1**–**K6**) ovules in KH at 5, 10, 20, 30, 40 and 50 days after pollination, respectively; en: endosperm; em: embryo; es: embryo sac; efn: endosperm free nuclei; ec: endosperm cells; ov: ovule; fne: free nuclear endosperm; fnp: free nuclear proembryo; hse: heart-shaped embryos; bar = 100 μm.

At 5–10 days after pollination, clear free nuclear endosperm was observed in the embryo sac of CK, DH and KH treatments, and the ovules developed normally. At 20 days after pollination, the free nuclear endosperm began to divide and the proembryo was observed in the embryo sac in CK treatment, whereas the embryo and the division of the free nuclear endosperm were not observed in the vast majority of embryo sacs in DH and KH treatments. The external morphology of normal seeds in CK treatment was significantly larger than that of aborted seeds in the DH and KH treatments, and the smaller ovules were aborted. At 30 days after pollination, in CK treatment, the free nuclear endosperm divided continuously around the embryo sac, the cellularized endosperm cells increased centripetally, the zygote divided to form a multicellular proembryo, and the endosperm nuclei divided vigorously. In contrast, in DH and KH treatments, the free nuclear endosperm around the embryo sac stopped dividing, embryo abortion was found in most of the embryo sac and the embryo sac appeared hollow. The diameter of the ovules in CK treatment was larger than most of those in the DH and KH treatments and the endosperm in DH and KH treatments was degraded. During 40–50 days after pollination, the endosperm gradually filled up the whole embryo sac, heart-shaped embryos were observed in CK treatment and embryo sac disintegration and severe atrophy of most of the ovules were observed in DH and KH treatment. Embryo and endosperm abortion were the main types of seed abortion in DH and KH treatments (Figure 8).

### 2.7. Endogenous Hormone Contents

**Figure 9:**
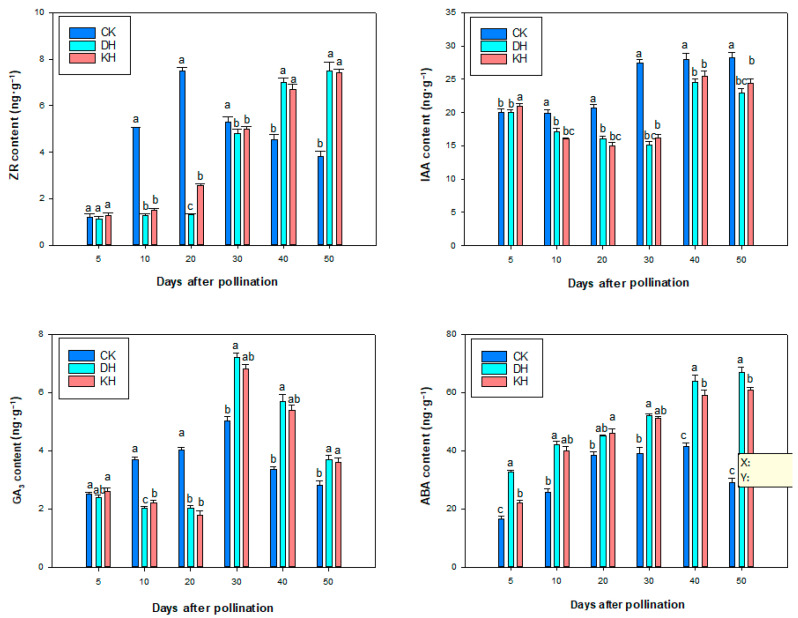
Changes in ZR, IAA, GA_3_, ABA contents during seed development in control (CK), stigmas treated with KCl solution before pollination (KH) and distant hybrid pollination (DH) treatments. Error bars represent mean ± standard deviation (SD); different lowercase letters indicate significant differences at *p* < 0.05.

The total GA_3_ content is relatively low, while the ABA content is high during seed development. In the CK treatment, the contents of ZR, GA, and ABA showed a dynamic trend of increasing and then decreasing from 0 to 50 days of embryo development, with peaks observed at 20, 30, and 40 days, respectively, while the IAA content showed a gradually increasing trend. The IAA content in the CK treatment was significantly higher than that in the DH and KH treatments from 10 to 50 d after pollination (Figure 9). In DH and KH treatments, the GA_3_ content decreased to its lowest values of 2.01 ng·g^−1^ and 1.77 ng·g^−1^ at 20 days after pollination, and then increased sharply to its highest values of 5.03 ng·g^−1^ and 7.21 ng·g^−1^ at 30 days after pollination, and then decreased sharply from 30 to 50 days after pollination. DH and KH treatments showed a gradual increase in ABA, and ZR contents after pollination, reaching their maximum values at 50 days after pollination, respectively. The contents of IAA, ZR and GA_3_ from most of the small ovules in DH and KH treatments were significantly lower than in CK during 10–20 days after pollination, while the ABA level was significantly higher than CK. At this time, the external morphology of normal seeds in CK was significantly larger than that of aborted seeds in DH and KH, indicating that the low content of IAA was unfavorable for hybridization seed development.

### 2.8. Nutrient Contents

**Figure 10:**
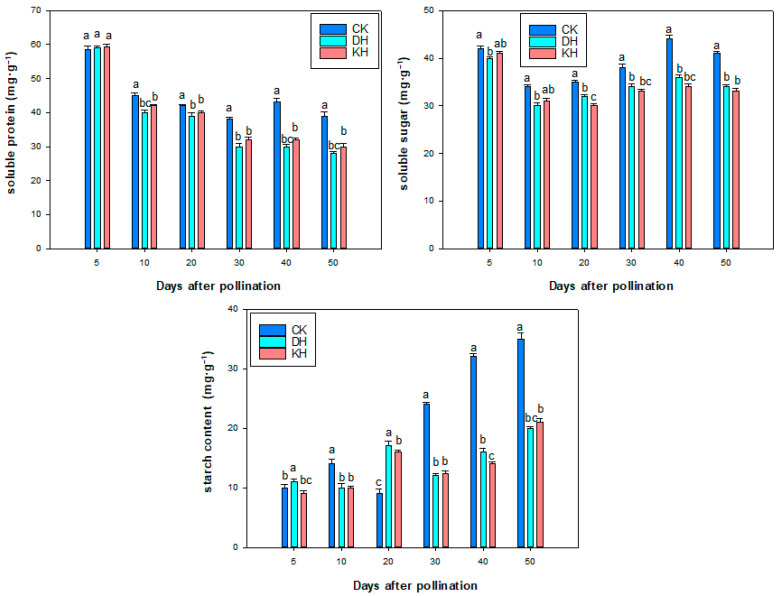
Soluble protein, soluble sugar, and starch contents during seed development in control (CK), stigmas treated with KCl solution before pollination (KH) and distant hybrid pollination (DH) treatments. Error bars represent mean ± standard deviation (SD); different lowercase letters indicate significant differences at *p* < 0.05.

Soluble protein levels changed significantly during seed development. In CK, the amount of soluble protein decreased continuously as the growing embryo consumed energy. The soluble protein content reached its minimum value of 38 mg·g^−1^ at 30 days after pollination. Subsequently, the soluble protein content increased to 43.25 mg·g^−1^ at 40 days after pollination and then decreased. The soluble protein in DH and KH treatments gradually decreased between 5 and 50 days after pollination, and the content remained lower than that in CK treatment (Figure 10).

The changes in soluble sugar content in CK, DH and KH treatments all showed a unimodal curve (Figure 10). As the growing embryo consumes energy, the level of soluble sugars in CK, DH and KH treatments continues to decrease, reaching its lowest values of 34.03, 30.2 and 31.15 mg·g^−1^ at 10 days after pollination, and then reaching its highest values at 40 days after pollination, with soluble sugar contents of 44.06, 36.12 and 34.20 mg·g^−1^, respectively. During embryonic development, the soluble sugar content of the CK treatment is consistently higher than that of DH and KH treatments (Figure 10).

In the CK, DH, and KH treatments, the starch content first increased, then decreased and finally increased rapidly. The starch content of CK reached its peak 10 days after pollination, then decreased to its lowest point of only 9.23 mg·g^−1^ at 20 days after pollination, and then rapidly increased to 35.50 mg·g^−1^ at 50 days after pollination. However, DH and KH treatments showed a peak in starch content at 20 days after pollination, and then decreased to 12.3 mg·g^−1^ and 12.06 mg·g^−1^ at 30 days after pollination, and then rapidly increased to reach its maximum values of 21.35 mg·g^−1^ and 20.25 mg·g^−1^ at 50 days after pollination, respectively. The starch content in CK was higher than that in DH and KH treatments except for 5 and 20 days after pollination (Figure 10).

## 3. Discussion

Pollen tube abnormality is a common cause of fertilization failure in distant hybridization, as well as the major factor contributing to pre-fertilization barriers and low seed set rates in plants. In fact, the accumulation of callose at the apex is considered to be a manifestation of pollen growth inhibition [40]. The main reasons for fertilization failure in crosses between *Cucurbita moschata* and *C. pepo* are callus formation at the end of the pollen tube and slow pollen tube development [41]. Our results showed that the stigma produced a considerable amount of callose, the number of pollen germinations was low and the pollen tubes showed tangled growth with swollen terminals in DH pollination. This phenomenon was also observed in *Solanum lycopersicum*, Rhododendron, Chrysanthemum, *Nicotiana tabacum*, and *hazelnut* spp. [42,43,44]. In the incompatible combination of *Chaenomeles japonica*, the pollen tube grows slowly and usually stagnates in the style, with heavy callose deposition at the tip of the tube [45]. Pollen grains in CK and KH treatments germinated 2 h after pollination, which is 2 h earlier than DH; pollen tubes in CK and KH entered the ovary 24 h after pollination, 12 h earlier than those in DH. This is in consistent with the results of Fu et al. [46], who found that salt treatment significantly increased the exposed surface area of papilla cells, the growth rate of pollen tube, and the number of pollen tubes reaching the ovule compared to CK. Our results indicated that the KCl solution had a good effect on the pollen–pistil compatibility between *P. ostii* ’Fengdan’ and *P. ludlowii*. In particular, the pollination method after treating the pistil with 1 mg·L^−1^ KCl solution was proved to be advantageous for pollen germination and subsequent pollen tube growth rate. We speculate that the plump papilla cells increased the contact area with the pollen grains. The exudate dissolved by the KCl solution reduced the rejection reaction of recognition proteins to foreign pollen grains, thus improving the energy supply for pollen growth.

ROS in plants affect the biotic and abiotic stress responses [47], and they regulate the pollen–stigma interaction in water lily [48,49]. SOD is one of the key antioxidant enzymes that directly regulate ROS accumulation [50]. Díaz et al. [51] observed that H_2_O_2_ contributes to the hardening of the cell wall, which limits cell elongation. In the present study, O_2_^−^, H_2_O_2_ and ^•^OH activities in DH pistils were significantly higher than those in KH and CK pistils during 4–12 h after pollination. During this period, the tubes’ growth pattern changed from curved to tangled growth, resulting in disordered and directionless growth, which prevented pollen tubes from growing polarly into the ovary. In both CK and KH treatments, a significant number of pollen tubes grow polarized towards the ovule direction at this time. At 24 h post-pollination, the pollen tubes in both CK and KH penetrated the ovary, whereas in DH, the pollen tubes failed to reach the ovary within this time frame. This suggests that a notable increase in the levels of the O_2_^−^ and H_2_O_2_ hinders the growth rate of the *P. ludlowii* pollen tubes, whereas KCl reduces the levels of ROS and contributes to pollen tube growth.

Exogenous hormones affect pollen germination [52]. IAA, ABA and GA promoted pollen germination and accelerated petunia pollen tube growth [53]. GA_3_ favored pollen germination and growth in tree peony [38,54]. Exogenous MeJA at a low concentration (0.05 mM) accelerated pollen tube growth in black pine, but a high concentration inhibited pollen tube growth [55]. BR significantly increased the pollen germination rate in *Arabidopsis thaliana* [56]. In this study, the levels of GA_3_ and BR in CK were significantly higher than those in DH and KH at 2–24 h after pollination, while the levels of MeJA and ABA were significantly lower in CK than those in DH and KH and faster elongation of pollen tubes was observed in CK compared to that observed in DH and KH. The higher levels of ABA and MeJA and the lower levels of GA_3_ and BR had a negative effect on the growth rate of pollen tubes in the pistils of *P. ostii* ‘Fengdan’, indicating that higher levels of GA_3_ and BR were beneficial for pollen tube growth in tree peonies [38,54].

Yang et al. [57] found that high levels of MDA were associated with cross-incompatibility and recognition barriers between pollen and stigma in distant hybridization between plum and apricot. In the poplar hybridization experiment, the SOD activities in the stigma of high-affinity pollination combinations were generally higher than those of low-affinity pollination combinations, while the MDA content showed the opposite trend [58]. Xu et al. also found that the increase in MDA content in tea (*Camellia sinensis*) was usually accompanied by a slowing of the pollen tube growth rate [59]. Liu et al. also found that high SOD and POD activities were beneficial for early pollen development in a sterile wheat line [60]. In this study, MDA levels in DH increased significantly during 4–12 h after pollination, accompanied by pollen tube twisting and curling. The SOD activity in CK and KH pistil was higher than that in DH pistil during 4–24 h after pollination, and the pollen tubes in CK and KH grew into the ovary at 24 h after pollination, whereas the pollen tube in the ovary was not observed until 48 h after pollination, indicating that increasing MDA content and decreasing SOD activity were not conducive to the growth of *P. ludlowii* pollen tube. These results are in accordance with Gao et al. [61], who found that high MDA levels were detrimental to pollen tube elongation in pear.

Embryo abortion in distant hybridization between groups is mainly due to the distant genetic relationship and the incompatibility and stable combination of parental genomes [8,62,63,64]. Chen et al. [65,66] also found that 10–30 days after flowering was the period of peak fruit growth and the main period of seed abortion in *P. ludlowii*. The abnormal stagnation of embryo development is the main reason for abortion seed abortion in *Syringa microphylla*, *Ziziphus jujube*, *Osmanthus fragrans*, and wild tomato species [67,68]. The seed abortion can be divided into two types: embryo and endosperm abortion and female infertility. Abnormalities in early embryo development were observed in DH and KH treatment, and embryo abortion mainly occurred between 10 and 30 days after pollination during the development of the pre-embryo. Cessation of embryo and endosperm development contributed to the seed abortion and the abnormally lower seed set in DH treatment and embryo and endosperm abortion was the main type of seed abortion in the DH treatment. This finding is in line with He et al., who found that early embryos often wilt and die shortly after fertilization in remote hybridization between *P. suffruticosa* and *P. lactiflora* [34]. KH treatment can significantly improve seed set, suggesting that this method may overcome the obstacle of distant hybridization between *P. ostii* and *P. ludlowii*. The setting rate of the male parent affects the embryo abortion rate of the hybrid offspring [69]. The genetic relationship between *P. ludlowii* and *P. lutea* is close. *Paeonia lutea*, which has a low seed abortion rate, has been used to improve the genes of cultivated peonies [70], resulting in popular varieties such as ‘High Noon’. The zygote and primary endosperm nucleus in *P. ludlowiii* were mainly aborted at 9–20 days after flowering and the number of aborted ovules in the ovary was more than 69% [71], suggesting that the possible reason for embryo abortion in *P. ludlowii* is the abnormality of fertilized embryos and primary endosperm nuclei caused by genetic defects. Is this responsible for the occurrence of fertilization disorders in crosses between *P. ludlowii* and cultivated peonies? More research is needed.

Dynamic changes in the content of endogenous hormones during ovule development and their proportional balance between them are closely related to ovule abortion [68,72]. During the main period of embryonic septation in *Hibiscus syriacus*, the content of GA_3_ decreased sharply, while the content of ABA increased rapidly. However, the low content of IAA was unfavorable for seed development and plump seed formation [68]. The content of growth-promoting endogenous hormones (GA_3_, IAA) was lower than that of normal ovules, and the content of growth-suppressing endogenous hormones (ABA) was significantly higher than that of normal ovules in the aborted ovules of *Robinia pseudoacacia* after 15 days of corolla unfolding [72]. In this study, we found that from 10 to 20 days after pollination, the contents of IAA, GA_3_ and ZR in CK-treated normal ovules were higher than those in DH- and KH-treated aborted ovules, while the levels of ABA were lower than those in DH- and KH-treated aborted ovules, which was similar to the results of the above study. This suggests that the low levels of IAA, GA_3_ and ZR and the high levels of ABA in the ovules of peony interspecific hybrids may be detrimental to the normal ovule development.

In the process of ovule development, changes in soluble sugars, starch, soluble proteins, enzyme activities and endogenous hormones are related to the biological functions and physiological activities of the cells, and sugars have osmo-regulatory and catalytic roles in ovule development, in addition to their role as an energy substance [65,67,68]. Aborted maize kernels were found to have lower levels of soluble sugars and starch than normal kernels [35]. In this study, the soluble sugar content of normal ovules treated with CK increased continuously from 10 to 20 days after flowering, whereas that of aborted ovules in DH and KH treatments increased and then decreased. It is suggested that the early abortion of peony ovules treated with DH and KH pollination may be related to the decrease in soluble sugar content of the ovules. Previously, it was found that in addition to the accumulation of organic matter, the weak capacity for organic matter synthesis is also an important physiological cause of maize seed abortion [35]. In the current study, from 10 to 30 days after flowering, the starch content of DH- and KH-treated aborted ovules increased but was still lower than that of normal ovules, indicating that the ability of starch conversion to soluble sugars in aborted ovules was weaker than that of CK-treated normal ovules, and the ability of starch synthesis in DH- and KH-treated aborted ovules was weaker than that of normal ovules. Soluble protein is an important nutrient for embryo development, which can activate cellular metabolic processes and promote embryo morphogenesis [68,73]. We found that the soluble protein content of DH and KH-treated aborted peony ovules was significantly lower than that of CK-treated normal ovules at 10–30 days after pollination, suggesting that the reduction in soluble protein content in the ovules may also lead to their abortion.

There are nine species of wild tree peony. Cultivated tree peonies (*P. suffruticosa*) that originated from homoploid hybridization include *P. qiui*, *P. jishanensis*, *P. ostii*, *P. cathayana*, and *P. rockii* [37,74]. Zhou et al. [74] and Guo et al. [75] found that *P. qiui*, *P. jishanensis*, *P. ostii*, *P. cathayana*, *P. decomposita*, *P. rotundiloba* and *P. rockii* are closely related by comparing nuclear and chloroplast phylogenies, so hybrids between these seven wild tree peonies and *P. suffruticosa* are compatible [37]. *Paeonia ludlowii* is relatively close in genetic relationship to *P. lutea*, but distantly related to the above seven wild tree peonies [37,74,75], so the interspecific hybrids between *P. lutea* and *P. ludlowii* showed some compatibility [37]. The genome of *P. ludlowii* is characterized by frequent chromosome reductions and centromere rearrangements [76], which is significantly different from that of *P. ostii* [77]. This may be the genetic reason for the fertilization barrier between *P. ludlowii* and *P. ostii.*

## 4. Material and Methods

### 4.1. Plant Materials and Pollen Collection

Ten-year-old *P. ostii* ‘Fengdan’, grown at the Peony Resource Center of Henan Institute of Science and Technology, was used as the female parent in the experiment. *Paeonia ludlowii* served as the male parent, and its fresh pollen grains were collected in May of the year before the experiment from Xizang College of Agriculture and Animal Husbandry, Linzhi, Xizang Province. *Paeonia ludlowii* pollen had a germination rate of 70.50 ± 2.25% after 24 h of drying in a 30 °C air-blast oven, followed by storage at 2 °C for about one year.

### 4.2. Sexual Cross Using Different Pollination Method

Three pollination methods including control (CK), stigmas treated with KCl solution before pollination (KH) and distant hybrid pollination (DH) were tested in the interspecific crossing experiment. For each of the three pollination methods, 300 well-developed *P. ostii* ‘Fengdan’ unopened flower buds were manually emasculated and placed in a pollination bag 3 days before flowering, and pollination was performed after approximately 3 days after emasculation. For CK, artificial pollination was performed with fresh pollen from *P. ostii* ‘Fengdan’. For KH, *P. ostii* ‘Fengdan’ stigmas were treated with 1.0 mg·L^−1^ KCl solutions and pollinated with *P. ludlowii* pollen grains 0.5 h later. For DH, *P. ostii* ‘Fengdan’ stigmas were pollinated with *P. ludlowii* pollen grains. CK, KH and DH pollinations were carried out at the full flowering stage of *P. ostii* ‘Fengdan’ in March 2023. For each of the three pollination methods, approximately the same amount of pollen was dispersed on different stigmas. At least 300 flowers of *P. ostii* ‘Fengdan’ were pollinated for each treatment.

### 4.3. Paeonia ostii ‘Fengdan’ Stigma Sampling and SEM Observation

Six stigma samples were collected from CK, KH, and DH treatments at 2 and 12 h after pollination, stigma samples were prepared according to the method of Jia et al. [78], and sample photos were taken using the Quanta 200 scanning electron microscope (FEI, Hillsboro, OR, USA).

### 4.4. Observation of Pollen Germination, Growth and Embryo Development

Pistils were collected from CK, KH, and DH at 0, 2, 4, 6, 8, 12, 24, 36, 48, 72, and 96 h after pollination, and then the trichomes were scraped off the surface of the pistil with a blade, and the pistil samples were immediately fixed in Carnot’s solution for 24 h. After washing three times with distilled water, the samples were processed according to the method of Wang et al. [68] and then pollen germination and growth in the pistil were observed under a Nikon ECLIPES Ci-S fluorescence microscope (Nikon, Tokyo, Japan) at a wavelength of 340~380 nm.

Developing ovary samples were collected at 5, 10, 20, 30, 40 and 50 days after pollination and 7–11 μm thick sections of ovaries were prepared by the paraffin sectioning method and stained with hematoxylin–eosin to examine embryonic development [65,79]. Six replicates were performed for each treatment.

### 4.5. O_2_^−^, H_2_O_2_, ^•^OH, SOD and MDA Level Analyses

Styles from CK, KH, and DH treatments were sampled to determine the levels of O_2_^−^, H_2_O_2_, ^•^OH, SOD and MDA at 2, 4, 6, 8, 12, 24, 36, and 48 h after pollination. O_2_^−^, ^•^OH, and H_2_O_2_ were determined following the method of Gajewska et al. [80], Gokce et al. [81], Cheeseman and Mukherjee et al. [82,83]. MDA level and SOD activity were determined following the method of Priyanka et al. [84] and Costa et al. [85], respectively. Six replicates were performed for each treatment.

### 4.6. Endogenous Hormone Content and Nutrient Content Analyses

Stigmas at 2, 4, 6, 8, 12, 24, 48, 72 and 96 h after pollination from CK, KH, and DH treatments, normal ovaries from CK treatment and abnormal ovaries from KH and DH treatments at 5, 10, 20, 30, 40 and 50 days after pollination were sampled to measure the endogenous hormone levels and nutrient levels. The levels of BR, ZR, IAA, GA_3_ and ABA were determined by enzyme-linked immunosorbent assay (ELISA) at Beijing tianyi Co, Ltd. (Beijing, China) following the manufacturer’s instructions [86]. Soluble protein, soluble sugar and starch contents were determined according to the method of Li et al. [87]. Six replicates were performed for each treatment.

### 4.7. Statistical Analysis

All data were processed and calculated using Microsoft Excel 2016 (Microsoft, Redmond, WA, USA). The data difference significance was calculated using Duncan’s multiple comparisons in SPSS version 19 (IBM, Chicago, IL, USA).

## 5. Conclusions

In this paper, we investigated the characteristics and reasons for the lack of crossability between *P. ludlowii* and *P. ostii* ‘Fengdan’ by microscopic observation and measurement of physiological indices in pistils. Swollen stigma papilla cells, high levels of H_2_O_2_, O_2_^−^, MDA, ^•^OH, ABA, and MeJA, and lower levels of BR and GA_3_ have a negative effect on pollen germination and elongation in the pistil of *P. ostii* ‘Fengdan’. A low pollen germination rate and pollen tube growth abnormality are the causes of fertilization failure in distant hybridization between *P. ludlowii* and *P. ostii* ‘Fengdan’, nutrient deficiencies, imbalances in phytohormones and antioxidant enzyme activities, and high contents of ABA during seed development resulted in seed abortion and decreased fruit set. Poor pollination, embryo and endosperm abortion may be the primary reason for seed abortion in DH treatment. The primary cause of cross failure between *P. ludlowii* and *P. ostii* ‘Fengdan’ is the pre-fertilization barrier. KH treatment may favor pollination and fertilization in distant hybridization between *P. ostii* ‘Fengdan’ and *P. ludlowii* and result in improved seed set. Our findings offer both practical and theoretical recommendations for pollination and production of high-quality tree peony varieties.

## Figures and Tables

**Table 1:** Effects of control (CK), stigmas treated with KCl solution before pollination (KH) and distant hybrid pollination (DH) treatments on seed set rates.

Pollination Methods	Seed Number per Fruit	Numbers of Plump Seeds per Fruit	Signal Follicle Weight
CK	20.90 ± 0.63 a	20.71 ± 1.20 a	9.80 ± 0.73 a
DH	11.89 ± 0.77 c	0.15 ± 0.10 c	1.82 ± 0.15 c
KH	18.52 ± 0.55 b	3.53 ± 0.35 b	3.68 ± 0.42 b

Note: Different lowercase letters indicate significant differences at *p* < 0.05, which is the same below.

## Data Availability

Data are contained within the article.
